# 91. Clinical Efficacy Endpoints from the Phase 3 CANOPY Study Evaluating Pemivibart

**DOI:** 10.1093/ofid/ofae631.028

**Published:** 2025-01-29

**Authors:** Myra Popejoy, Anna Holmes, Chloe Katz, Anne-Marie Phelan, Kazima Tosh, Yong Li, Deepali Gupta, Pamela Hawn, Kristin Narayan, Kathryn Mahoney, Mark Wingertzahn

**Affiliations:** Invivyd, Inc., Waltham, Massachusetts; Invivyd, Inc., Waltham, Massachusetts; Invivyd, Inc., Waltham, Massachusetts; Invivyd, Inc., Waltham, Massachusetts; Invivyd, Inc., Waltham, Massachusetts; Invivyd, Inc., Waltham, Massachusetts; Invivyd, Inc., Waltham, Massachusetts; Invivyd, Inc., Waltham, Massachusetts; Invivyd, Inc., Waltham, Massachusetts; Invivyd, Inc., Waltham, Massachusetts; Invivyd, Inc., Waltham, Massachusetts

## Abstract

**Background:**

Pemivibart (PEM) is a half-life extended recombinant human monoclonal IgG1 antibody that targets the SARS-CoV-2 spike protein receptor binding domain. PEM has been granted emergency use authorization (EUA) for the pre-exposure prophylaxis of COVID-19 in certain adults and adolescents with moderate-to-severe immune compromise.

**Figure:**
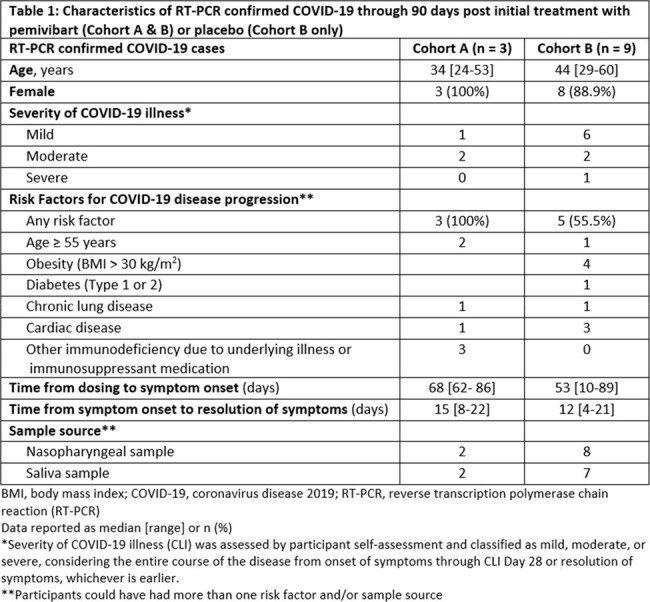

**Methods:**

CANOPY (NCT06039449) is a Phase 3 study investigating the prevention of COVID-19 in immunocompromised participants (ppts) (Cohort A; single arm, open label) and in ppts at risk of exposure to SARS-CoV-2 (Cohort B; randomized 2:1 PEM 4500 mg or placebo). Ppts received an initial dose of study drug administered via intravenous (IV) infusion on Day 1 followed by a second dose of study drug approximately 3 months later. The safety, tolerability, pharmacokinetics (PK), immunogenicity, and clinical efficacy of PEM is being evaluated. Here we describe an exploratory endpoint of reverse transcription polymerase chain reaction (RT-PCR)-confirmed symptomatic COVID-19 that developed within 90 days following the first administration of either PEM or placebo among ppts without SARS-CoV-2 infection at baseline. Nasopharyngeal and saliva samples were collected for central SARS-CoV-2 testing by RT-PCR and subsequent whole genome sequencing (WGS).

**Results:**

RT-PCR-confirmed COVID-19 cases were observed in 3 of 298 ppts (1%) with significant immune compromise who received a full initial dose of PEM in Cohort A and 9 of 484 (1.9%) ppts who received either PEM or placebo in Cohort B. In Cohort B, RT-PCR confirmed COVID-19 cases were observed in 1 (0.3%) PEM-treated ppt compared to 8 (5.0%) placebo-treated ppts (Absolute Risk Reduction: -4.73% (95% CI: -8.2, -1.3), nominal p = 0.0070). Characteristics of these cases are described in Table 1. All but one COVID-19 case were self-reported as mild or moderate in severity. SARS-CoV-2 variants including JN.1, EG.5.1, and other related lineages were identified.

**Conclusion:**

This exploratory analysis of COVID-19 events demonstrated a clinically meaningful reduction in COVID-19 with PEM treatment versus placebo.

**Disclosures:**

**Myra Popejoy, Pharm.D.**, Invivyd, Inc.: employee|Invivyd, Inc.: Stocks/Bonds (Public Company) **Anna Holmes, PhD**, Invivyd, Inc.: Employee|Invivyd, Inc.: Stocks/Bonds (Public Company) **Chloe Katz, PMP**, Invivyd, Inc.: Employee|Invivyd, Inc.: Stocks/Bonds (Public Company) **Anne-Marie Phelan, MA**, Invivyd, Inc.: Employee|Invivyd, Inc.: Stocks/Bonds (Public Company) **Kazima Tosh, Ph.D.**, Invivyd, Inc.: Employee|Invivyd, Inc.: Stocks/Bonds (Public Company) **Yong Li, PhD**, Invivyd, Inc.: Employee|Invivyd, Inc.: Stocks/Bonds (Public Company) **Deepali Gupta, BSc**, Invivyd, Inc.: Employee|Invivyd, Inc.: Stocks/Bonds (Public Company) **Pamela Hawn, Pharm.D.**, Invivyd, Inc.: Employee|Invivyd, Inc.: Stocks/Bonds (Public Company) **Kristin Narayan, Ph.D.**, Invivyd, Inc.: Employee|Invivyd, Inc.: Stocks/Bonds (Public Company) **Kathryn Mahoney, PharmD**, Invivyd, Inc.: Employee|Invivyd, Inc.: Stocks/Bonds (Public Company) **Mark Wingertzahn, Ph.D.**, Invivyd, Inc.: Employee|Invivyd, Inc.: Stocks/Bonds (Public Company)

